# Prognostic roles of a novel basement membranes-related gene signature in lung adenocarcinoma

**DOI:** 10.3389/fgene.2023.1100560

**Published:** 2023-02-09

**Authors:** Xingzhuang Zhu, Xiaoyan Liu, Xiaowen Qiu, Zihao Niu, Wei Dong, Yipeng Song

**Affiliations:** ^1^ Department of Oncology, Qingdao University, Qingdao, China; ^2^ Department of Radiation Oncology, Yantai Yuhuangding Hospital, Affiliated Hospital of Qingdao University, Yantai, China; ^3^ Department of Oncology, Binzhou Medical University, Yantai, China; ^4^ Ministry of Education Key Laboratory of Otolaryngology Head and Neck Surgery, Department of Otolaryngology Head and Neck Surgery, Beijing Tongren Hospital, Capital Medical University, Beijing, China

**Keywords:** basement membranes, lung adenocarcinoma, TCGA, GEO, prognosis

## Abstract

**Background:** The basement membranes (BMs) are involved in tumor progression, while few comprehensive analyses to date are performed on the role of BM-related gene signatures in lung adenocarcinoma (LUAD). Thus, we aimed to develop a novel prognostic model in LUAD based on BMs-related gene profiling.

**Methods:** The LUAD BMs-related gene profiling and corresponding clinicopathological data were obtained from the basement membrane BASE, The Cancer Genome Atlas (TCGA) and gene expression omnibus (GEO) databases. The Cox regression and least absolute shrinkage and selection operator (LASSO) methods were used to construct a BMs-based risk signature. The concordance index (C-index), receiver operating characteristic (ROC), and calibration curves were generated to evaluate the nomogram. The GSE72094 dataset was used to validate prediction of the signature. The differences in functional enrichment, immune infiltration, and drug sensitivity analyses were compared based on risk score.

**Results:** In TCGA training cohort, 10 BMs-related genes were found, (e.g., ACAN, ADAMTS15, ADAMTS8, BCAN, etc). The signal signature based on these 10 genes was categorized into high- and low-risk groups regarding survival differences (*p* < 0.001). Multivariable analysis showed that the signature of combined 10 BMs-related genes was an independent prognostic predictor. Such a prognostic value of BMs-based signature in validation cohort of the GSE72094 were further verified. The GEO verification, C-index, and ROC curve showed that the nomogram had accurate prediction performance. The functional analysis suggested that BMs were mainly enriched in extracellular matrix-receptor (ECM-receptor) interaction. Moreover, the BMs-based model was correlated with immune checkpoint.

**Conclusion:** This study identified BMs-based risk signature genes and demonstrated their ability to predict prognosis and guide personalized treatment of patients with LUAD.

## Introduction

Lung cancer is the second most common cancer and causes a high mortality in the world (Abe and Tanaka, 2016). Lung adenocarcinoma (LUAD) was the predominant type of lung cancer, consisted of approximately 40% cases of lung cancer (Sung et al., 2021). Although great progress has been made in diagnosis and treatment of LUAD, especially in targeting and immunotherapy, the 5-year overall survival (OS) of patients with LUAD remains poor, with approximately 15% only (Wang et al., 2021). LUAD had high molecular heterogeneity and a tendency of early metastasis (Devarakonda et al., 2015). Current methods are still difficult to accurately predict the occurrence and prognosis of LUAD (Calvayrac et al., 2017). Therefore, there is an urgent need to develop more effective and reliable prognostic biomarkers to identify beneficiary patients.

The basement membranes (BMs) are the oldest animal extracellular matrix (ECM), forming a flaky structure that lies under the epithelial cells and surrounds most tissues (Pozzi et al., 2017). The respective planar networks of laminin and type IV collagen molecules are associated with cell surface interactions, providing a scaffold structure for building BMs along the tissue (Yurchenco, 2011). The BMs can not only be used to resist mechanical stress, determine tissue shape and create diffusion barriers, but also provide clues to guide cell polarity, differentiation, migration and survival (Jayadev et al., 2022). The variation of more than 20 BMs genes emphasizes the diversity and basic function of the BM (Nyström et al., 2017). BM protein expression and turnover defects are related to the occurrence of cancer (Naba et al., 2014).

The altered expression of ECM macromolecules in tumor microenvironment (TME) affects the growth, survival, adhesion and migration of cancer cells (Fares et al., 2020). Recently, the study has found that BMs play a critical role in the development of human diseases (Jayadev et al., 2022). For example, In the early development of breast cancer, cancer cells invade through the BM foramen, which is one of the key steps of metastasis (Sikic et al., 2022). At present, the research of BMs in LUAD is relatively few, and thus further research in this filed is needed.

Because the prediction of multi-gene model is better than that of single-gene one, we carried out this study (Srivastava and Gopal-Srivastava, 2002). In this study, we downloaded data from The Cancer Genome Atlas (TCGA) to build a BMs-related genes signature in order to predict the clinical outcome in LUAD patients. The predictive ability of the signature is then verified using data from the Gene Expression Omnibus (GEO). Finally, a risk prognosis model based on the BMs-related genes signature was established, which offered a more accurate prediction of LUAD prognosis than simple clinicopathologic nomograms.

## Materials and methods

### Data collection and determination of BMs differential expression

The RNA-seq data expression and clinical information of 59 normal lung and 539 LUAD tissues were obtained from TCGA database (https://portal.gdc.cancer.gov). The LUAD RNA-seq data of 398 cases were downloaded from GEO database (https://www.ncbi.nlm.nih.gov/geo/). After the data were integrated, 449 cases in TCGA database were used as training cohort, while 398 cases in GSE72094 database were used as validation cohort. BMs were downloaded from hallmark gene sets in the basement membrane BASE database (https://bmbase.manchester.ac.uk) (Jayadev et al., 2022). Different gene expression data sets were normalized by R software. The differentially expressed BMs were identified by “limma” package based on R software according to the criteria of | logFC | > 1 and false discovery rate (FDR) < 0.05.

### Construction and validation of a predictive model based on BMs

In the training cohort, univariate Cox regression analysis was used to analyze the differentially expressed BMs-related genes (*p* < 0.05). Then, the regression analysis of least absolute shrinkage and selection operator (LASSO) was used, and the candidate BMs-related genes with predictive significance were screened by “glmnet” R package. Next, the optimal weighting coefficient of each prognostic candidate BM gene was determined by multivariable Cox regression analysis. All differentially expressed and prognostically significant BM-related genes were included by BM features. This specific risk score is calculated by the following formula: (Coef1 expression *mRNA1) + (Coef2 expression *mRNA2) + (Coef n expression *mRNAn), where Coef is the corresponding coefficient of mRNA in the LASSO regression model.

According to the median of risk score, patients with LUAD were divided into high-risk group and low-risk group. To assess the prognosis of both groups, the OS was performed by the Kaplan-Meier curve. The prognostic ability of the risk model was evaluated by time-dependent ROC analysis using the “survival ROC” software package (Janssens and Martens. 2020). To verify the BM signature, use the risk score of LUAD cases in the GSE72094 dataset to verify the accuracy of the model. In order to verify the BM signature, the risk score of LUAD cases in the GSE72094 data set was used by the same method to verify the accuracy of the model.

### Establishment of a prognostic nomogram in LUAD

In TCGA training set, the association between BMs-related genes signature and clinical variables was performed. In addition, both univariate and multivariable Cox regression analysis were conducted to explore whether the risk score had an independent prognostic value in patients with LUAD. The probability of 1-year, 3-year and 5-year OS in LUAD patients were assessed by clinical variables and risk score. The accuracy of nomogram was performed to evaluated by concordance index (C-index) and calibration curve.

### Analysis of prognosis and immune value of BMs-related genes signature

The prognostic survival value of BMs-related genes signature mRNAs in LUAD was analyzed by Kaplan-Meier survival analyses. Then, mRNAs with high prognostic potential were chosen for the next stage of evaluation. The correlation between the immune function and immune cells of prognostic signature genes was analyzed and scored by ssGESA algorithm.

### Functional enrichment analyses and protein-protein interaction (PPI)

Gene ontology (GO) analysis, including molecular function (MF), biological processes (BP), and cellular components (CC), and the Kyoto Encyclopedia of Genes and Genomes (KEGG) pathway analysis was performed by the “clusterProfiler” package (Kanehisa and Goto, 2000). FDR and *p*-value < 0.05 were considered to be significantly enriched. By submitting the differentially expressed BMs information to the STRING database (http://www.string-db.org/), the protein-protein interaction information was obtained (Szklarczyk et al., 2019). The construction and visualization of PPI network was realized by the Cytoscape software (Otasek et al., 2019). According to the MCODE plug-in, we selected the MCODE score > 10 to filter out the most significant module in the PPI network.

### Immune cell infiltration analysis

We utlized a series of algorithms, including CIBERSORT, CIBERSORT-ABS, QUANTISEQ, MCP-counter, XCELL, TIMER, and EPIC algorithms, to evaluate the level of immune cell permeability between the high-risk group and the low-risk group according to the differentially expressed BMs-based signature (Newman et al., 2015). We explored the expression of several immune checkpoints, such as CD276, TNFSF9, CD200R1, CD28, CD80, CD48, TNFS18, TNFS15 and CD40LG, for immune checkpoint blockade therapy.

### Statistics analysis

All statistical analyses were performed using R software (version 4.1.3). Continuous variables are tested by the student T test, while classified variables are tested by chi-square test. A *p*-value < 0.05 was considered significant.

## Results

### Identification of a BM-related genes signature

**FIGURE 1:**
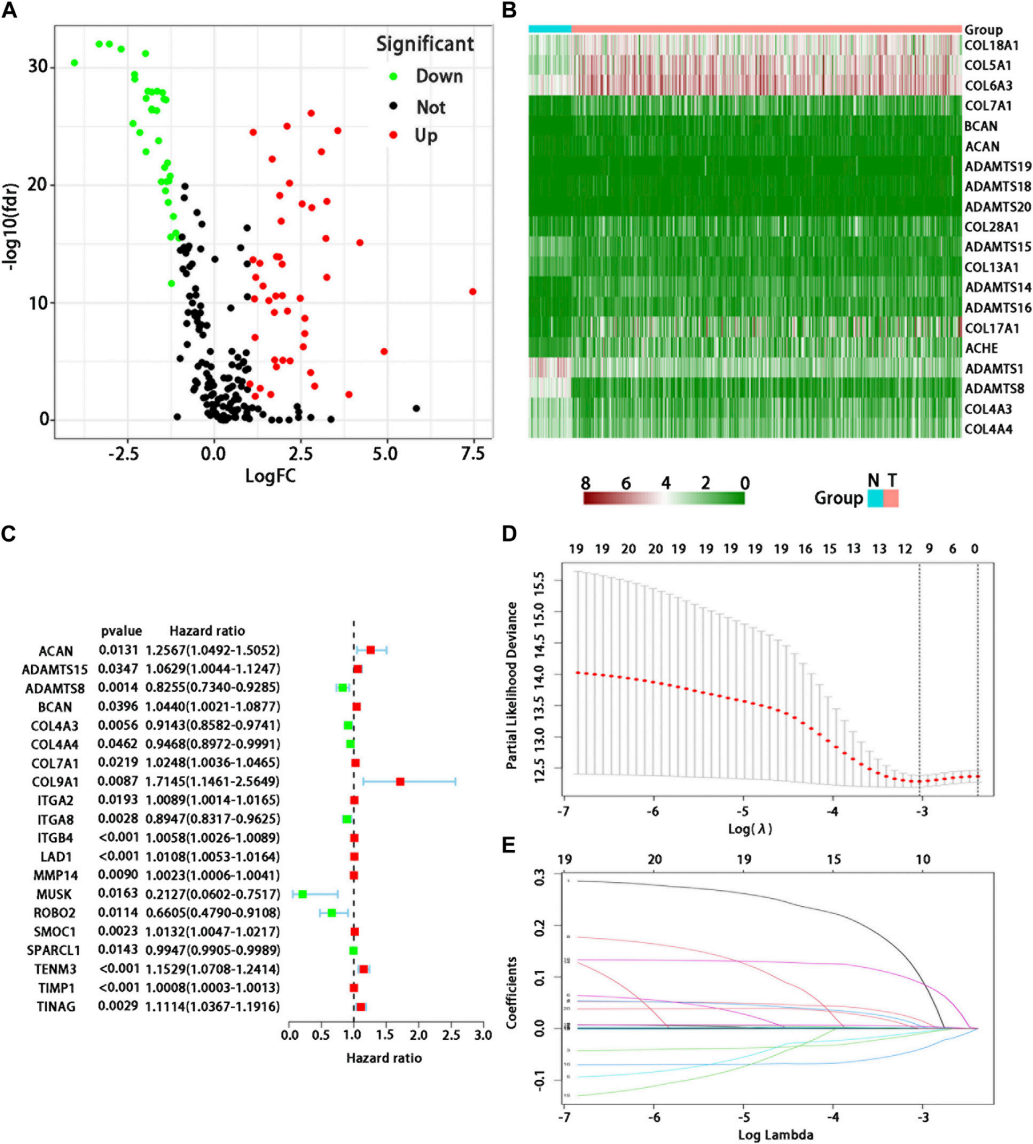
Analysis of differential expression and establishment of prognostic model of BMs-related genes in LUAD. **(A)** Volcano map showed differential expression BMs-related genes. Red dots represent upregulated BMs-associated mRNAs, green dots represent downregulated ones, and black dots represent mRNAs with no significant differential expression; **(B)** Heatmap showed differential expression BMs-related genes. Red represents high expression and green represents low expression. Acronym: N = normal tissue, T = tumor tissue. **(C)** Determination of prognostic BMs-related genes under univariate Cox regression analysis. **(D)** LASSO coefficient profiles of the 10 genes in LUAD. A coefficient profile plot was generated against the log (lambda) sequence. **(E)** Selection of the optimal parameter (lambda) in the LASSO model for LUAD.

In 539 tumor and 59 normal tissues, we found 81 differentially expressed BMs genes (*p* < 0.05, and logFC | > 1), including 47 up-regulated and 34 down-regulated differential expression ones (Figure 1A). The differential expression of each sample was shown in the heatmap (Figure 1B). After excluding 72 patients without appropriate follow-up or lack of important clinical information, 449 patients were included in the TCGA training set to identify the prognosis-related BMs genes for constructing a BMs-based signature. The clinicopathological information of LUAD in the TCGA database was shown in Table 1.

**TABLE 1:** Summary of the Clinicopathological characteristics of patients with LUAD.

Covariates	Group	Patient number (%)	*p*-value^1^	*p*-value^2^
Age	≤65	218 (48.6)	0.271	0.182
	>65	231 (51.4)		
Gender	Female	248 (55.2)	0.832	0.942
	Male	201 (44.8)		
Chemotherapy	Yes	181 (40.3)	0.091	0.209
	No	268 (59.7)		
Radiotherapy	Yes	106 (23.6)	4.20e-05	0.008
	No	343 (76.4)		
Stage	Stage I-II	358 (79.7)	4.28e-07	<0.001
	Stage III-IV	91 (20.3)		

1 *p*-value of univariate Cox regression.

2 *p*-value of multivariable Cox regression.

We used the univariate Cox regression to analyze individually the differentially expressed BMs gene profile, 20 BMs genes were found from TCGA training cohort (Figure 1C). Lasso regression analysis was carried out among 20 BMs genes, of which 10 BMs genes were found to be significant and selected as the BM signature candidates genes (Figures 1D, E). The multivariate Cox regression analysis was used to determine the corresponding regression coefficients of each candidate in this BM-related risk gene signature (Table 2). Finally, according to 449 LUAD cases in TCGA training cohort, the 10 BM-related risk gene signature was constructed, and the risk score was calculated based on the linear combination of gene expression levels and corresponding regression coefficients. Among them, the calculation formula of correlation coefficient among 10 BM-related genes was as following (Table 2): risk score= (0.1021 × ACAN expression) +(0.0162 × ADAMTS15 expression) + (−0.010 × ADAMTS8 expression) + (0.0058 × BCAN expression) + (−0.0070 × COL4A3 expression) + (−0.0405 × ITGA8 expression) + (0.0017 × ITGB4 expression) + (0.0043 ×LAD1expression) + (−0.0891 × TENM3 expression) + (0.0003 × TIMP1 expression).

**TABLE 2:** The 10 BMs-related gene list and coefficient.

Genes	Coefficient
ACAN	0.1021
ADAMTS15	0.0162
ADAMTS8	−0.010
BCAN	0.0058
COL4A3	−0.0070
ITGA8	−0.0405
ITGB4	0.0017
LAD1	0.0043
TENM3	0.0891
TIMP1	0.0003

### Prognostic value of BM-related risk gene signature in the training cohort

**FIGURE 2:**
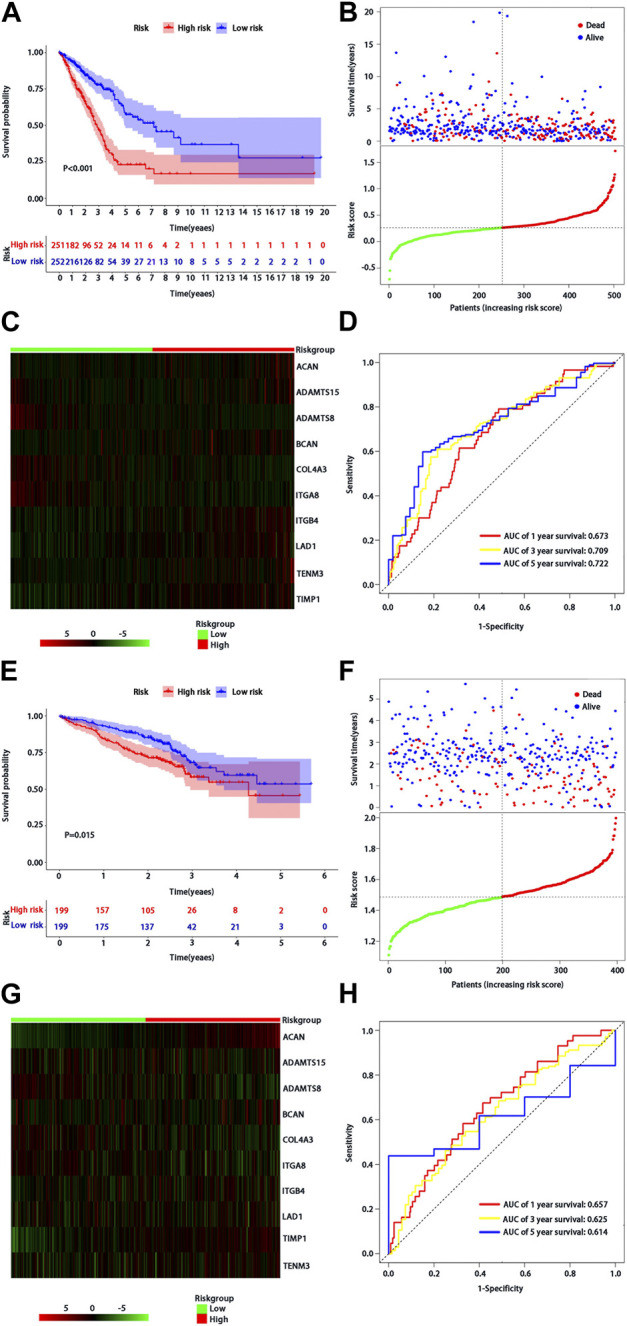
Assessment of BMs-related genes signature. **(A)** Kaplan-Meier survival analysis of patients with LUAD in high and low risk groups of TCGA training cohort; **(B)** Survival status distribution based on the median risk score in TCGA training cohort; Red represents high risk and green represents low risk; **(C)** Heatmap showed differential expression of BMs in high and low risk groups of TCGA training cohort; **(D)** ROC curve analysis of risk score predicting overall survival in TCGA training cohort. **(E)** Kaplan-Meier survival analysis of patients with LUAD in high and low risk groups of the GSE72094 validation cohort; **(F)** Survival status distribution based on the median risk score of the GSE72094 validation cohort; Red represents high risk and green represents low risk; **(G)** Heatmap showed differential expression of BMs in high and low risk groups in the GSE72094 validation cohort t; **(H)** ROC curve analysis of risk score predicting overall survival in the GSE72094 validation cohort.

According to the median of risk score, the patients with LUAD were divided into high- and low-risk groups. The LUAD patients in the low-risk group had significantly longer OS time than those in the high-risk group (*p* < 0.001) (Figure 2A). From the distribution of risk score (Figure 2B), the number of deaths in the high-risk group was significantly higher than that in the low-risk group. The heatmap showed the differential expression of these 10 BM-related risk genes between the low-risk group and the high-risk group (Figure 2C). The area under the time-dependent ROC curve at 1-, 3-, and 5 years was 0.673, 0.709, and 0.722 in the two groups of patients, respectively, indicating a good performance of the risk model for predicting the survival of LUAD patients (Figure 2D).

### Prognostic value of BMs-related genes signature in validation cohort

We used the same method to verify the prognostic value of BMs-based signature in GSE72094 verification cohort. The survival curve showed that the OS of patients in the low-risk group was better than that in the high-risk group (*p* = 0.015) (Figure 2E), and there were more deaths in the high-risk group than that in the low-risk group (Figure 2F). The expression profiles of 10-BMs between the low-risk group and the high-risk group were drawn in the heatmap (Figure 2G). Compared with the training cohort, the area under the time-dependent ROC of the validation cohort at 1-, 3-, and 5 years was 0.667, 0625, and 0.614, respectively, which also showed a good verification (Figure 2H).

### Prognostic significance and immune infiltration of differential expression BMs genes

**FIGURE 3:**
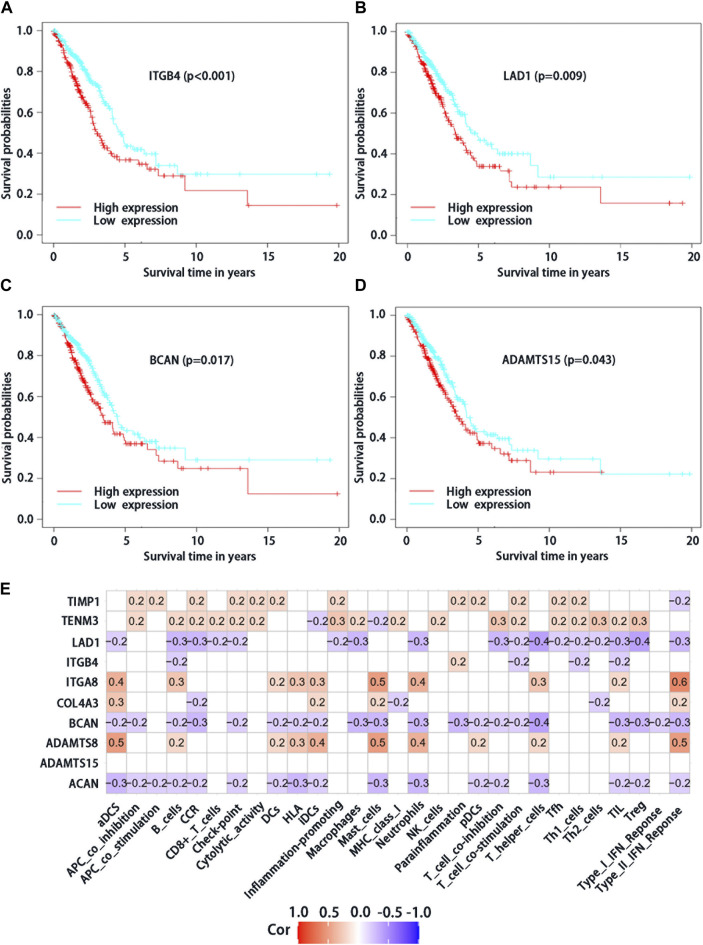
Analysis of survival significance of differential expression BMs-related genes signature in high-risk group and low-risk group including high expression of ITGB4 **(A)**, LAD1 **(B)**, BCAN **(C)** and ADAMTS15 **(D)**. The red curve represents the high expression of mRNA and the blue curve represents the low expression. **(E)** Analysis of immune cells and immunology functions associated with differential expression genes. Correlation analysis of differential expression genes with immune cells and immunology functions. The red color represents positive correlations, the blue color represents negative correlations, and the white indicates relationships without a statistical difference.

From the 10 BMs genes which were used to construct the risk score model, four of them based on survival significance were screened. After we draw the survival curve of these four genes, the high expression group of ITGB4 (*p* < 0.001), LAD1 (*p* = 0.009), BCAN (*p* = 0.017), and ADAMTS15 (*p* = 0.043) had a worse prognosis than the low expression group (Figures 3A–D), respectively, suggesting that the high expression of ITGB4, LAD1, BCAN and ADAMTS15 might be related to the progression of the tumor. Moreover, the correlation analysis of differential expression genes in immune cells and function showed that TIMP1, TENM3, ITGA8 and ADAMTS8 were positively correlated with most immune cells and immune function (Figure 3E), while ACAN, BCAN, and LAD1 were negatively correlated, suggesting that these genes may play significant roles in LUAD immunity and deserve further study. Taken together, these results suggested that above genes with differential expression could play a crucial role in the immune regulation of LUAD.

### Stratified analysis of association between BMs-based signature and prognosis by clinical features in patients with LUAD

**FIGURE 4:**
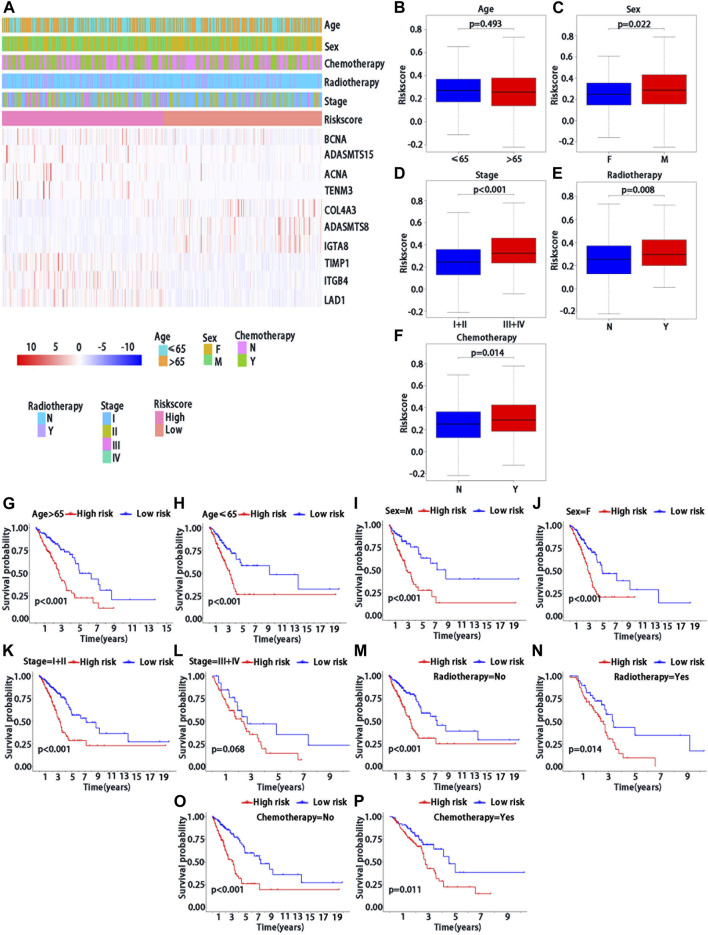
Clinical characteristics of BMs-related genes signature with LUAD. **(A)** The heatmap of clinical factors and BM genes in high and low-risk with LUAD. Differential expression in clinical factors of age**(B)**, sex **(C)**, stage **(D)**, radiotherapy **(E)** and chemotherapy **(F)** under high and low-risk with LUAD. Prognostic analysis of different clinical factors, including age>65 **(G)**, age≤65 **(H)**, sex = M **(I)**, sex = F **(J)**, stage = I + II **(K)**, stage = III + IV **(L)**, radiotherapy = No **(M)**, radiotherapy = Yes **(N)**, chemotherapy = No **(O)** and chemotherapy = Yes **(P)** in K-M survival analysis of high and low risk. Red curve represents the high-risk group and blue curve represents the low-risk group. Abbreviations: F = female, M = male, I + II = I and II stage, III + IV = III and IV stage.

We further confirmed the association between risk score and clinical characteristics of LUAD patients. From the heatmap (Figure 4A), we found that sex (*p* = 0.022), stage (*p* < 0.001), radiotherapy (*p* = 0.008) and chemotherapy (*p* = 0.014) had significantly statistical differences between the high-risk and low-risk groups, while the age (*p* = 0.493) had no significant statistical significance (Figures 4B–F). Thus, we performed the stratified analysis of risk score on survival by clinical factors. We found that there were significant survival differences between low-risk and high-risk groups in different subgroups with different clinical factors, including as age (≤65 *vs.* > 65 years) (*p* < 0.001), sex (male *vs*. Female) (*p* < 0.001), with chemotherapy (*p* = 0.011), without chemotherapy (*p* < 0.001), with radiotherapy (*p* = 0.014) and stage (I/II *vs.* III/IV) (*p* < 0.001), while no significant survival difference in patients without radiotherapy (*p* > 0.068) (Figures 4G–K).

### Multivariable analysis of prognosis of risk score in LUAD

**FIGURE 5:**
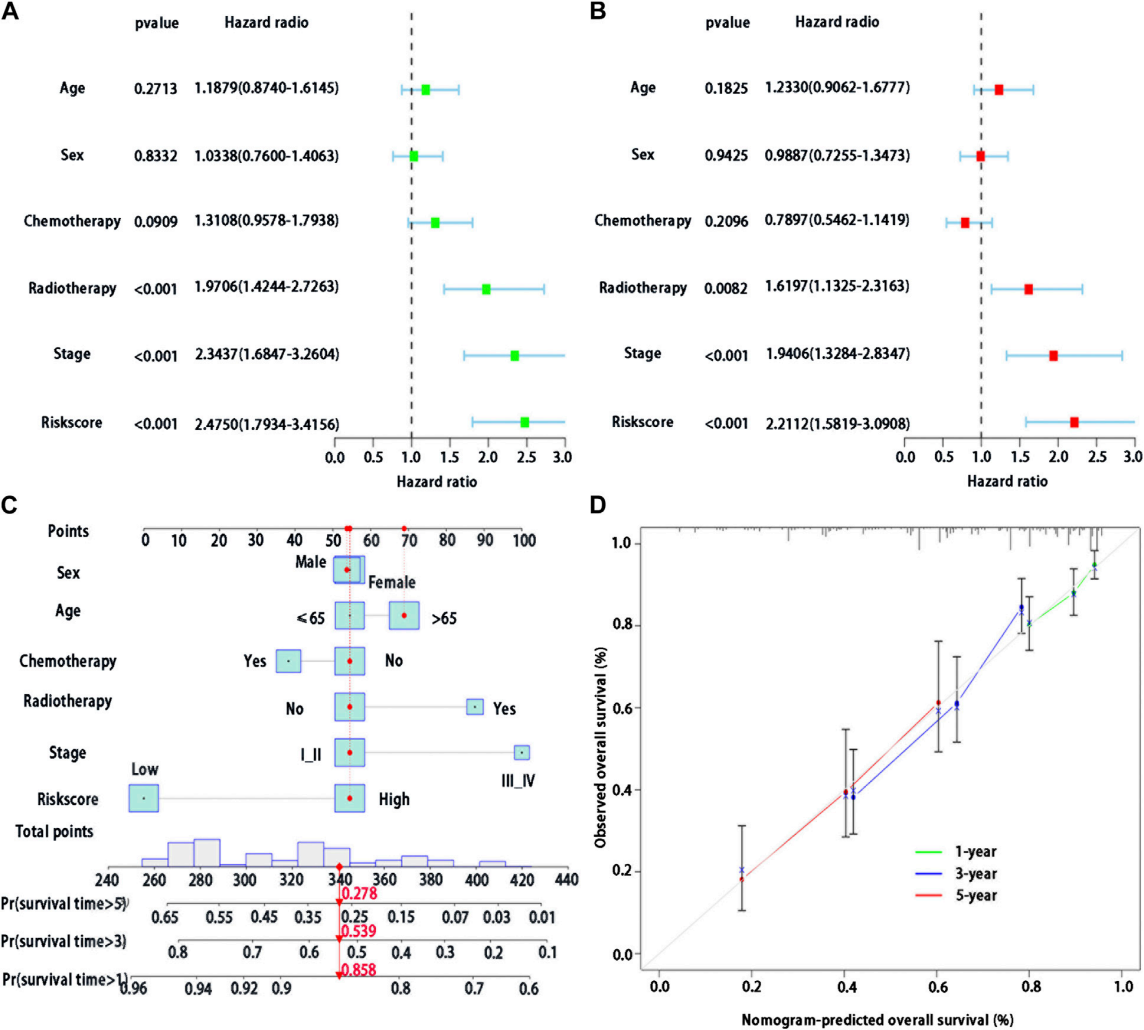
Development of a BMs-related genes signature nomogram with LUAD. **(A)** Univariate Cox regression analysis of the clinical features and the risk score in patients with LUAD; **(B)** Multivariate Cox regression analysis of the clinical features and the risk score in patients with LUAD. **(C)** Nomogram for BMs risk score and clinical features; **(D)** The calibration plots for predicting 1-,3- or 5-year survival probability.

Multivariable Cox regression analysis was used to analyze whether risk score could be regarded as independent prognostic indicators of LUAD after adjustment with other prognostic factors. We first found that risk score (*p* < 0.001), stage (*p* < 0.001), and radiotherapy (*p* < 0.001) were significantly associated with survival in patients with LUAD by the univariate analysis (Figure 5A). We then performed the multivariable Cox regression analysis to show a significant association between risk score and prognosis (*p* < 0.001) (Figure 5B), indicating that risk score may serve as an independent predictor of prognosis of patients with LUAD.

### Nomogram of BMs-related genes signature in patients with LUAD

In the training cohort, we used BMs-based signature risk score, age, sex, stage, radiotherapy and chemotherapy to develop a visual nomogram for 1-, 3- and 5-year individual survival prediction (Figure 5C). Bootstrap verification was performed to verify the accuracy of the nomogram. The C-index of the training cohort was 0.663, which showed that the nomogram had good prediction ability in LUAD. We then draw the calibration curve (Figure 5D) to verify the accuracy of the nomogram. The calibration curve showed that the survival probability of the actual observation and prediction was satisfactory in terms of 1-, 3- and 5-year consistency.

### Functional enrichment analysis and PPI of the BMs-related genes

**FIGURE 6:**
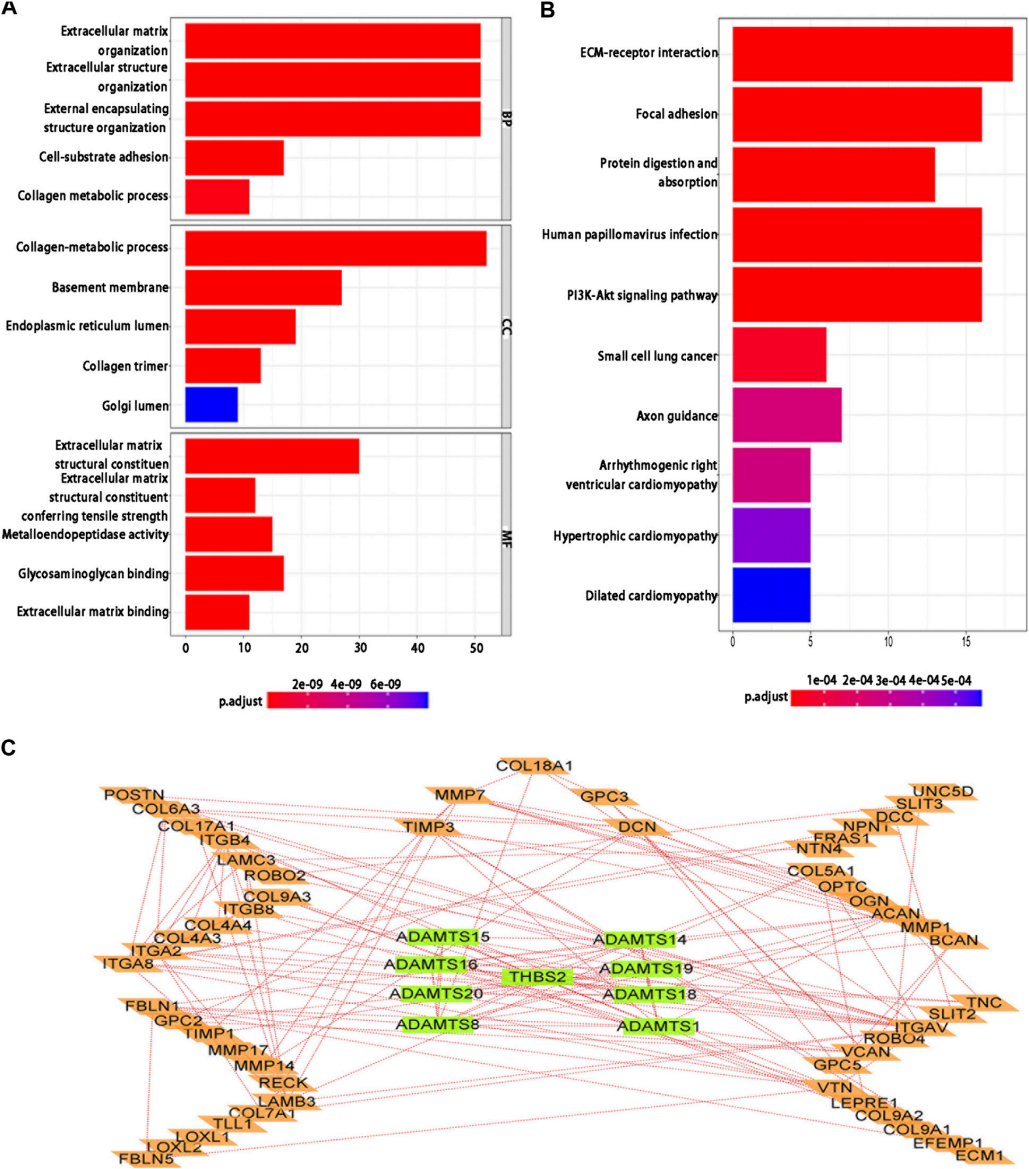
Differentially expressed BMs-related genes analysis of enrichment function in LUAD. **(A)** GO function analysis; **(B)** KEGG pathway analysis; **(C)** PPI network.

To investigate the function and potential pathway of BMs genes in LUAD, we performed the GO and KEGG analysis for differentially expressed BMs genes. Based on the results of biological process (BP)’s analysis, we found that 81 BMs genes were involved in extracellular matrix organization, extracellular structure organization, and external encapsulating structure organization. The cellular component (CC) analysis demonstrated that collagen-containing extracellular matrix and basement membrane. Molecular function (MF) analysis also showed that 81 BMs were mainly related to extracellular matrix structural constituent, extracellular matrix structural constituent conferring tensile strength, metalloendopeptidase activity, glycosaminoglycan binding, and extracellular matrix binding (Figure 6A). Moreover, the KEGG pathway enrichment analysis found that the main enrichment pathways of BMs differentially expressed genes included ECM-receptor interaction, focal adhesion, protein digestion and absorption, human papillomavirus infection, PI3K-Akt signaling pathway, small cell lung cancer, axon guidance, arrhythmogenic right ventricular cardiomyopathy, hypertrophic cardiomyopathy, and dilated cardiomyopathy (Figure 6B). From the STRING database, we found that the PPI network based on differentially expressed BMs genes was mainly composed of 62 nodes and 173 edges (Figure 6C).

### Analysis of immune checkpoint analysis for BMs-related genes signature in patients with LUAD

The relationship between risk score and immune checkpoint is worthy of our investigation as the immune checkpoints play important roles in immunotherapy. We found that there were differences in the expression of CD276, TNFSF9, CD200R1, CD28, CD80, CD48, TNFS18, TNFS15 and CD40LG between high-risk and low-risk groups. Among them, CD276, and TNFSF9 were highly expressed in the high-risk group (Supplementary Figures S1 A, B), while CD200R1, CD28, CD80, CD48, TNFS18, TNFS15, and CD40LG were highly expressed in the low-risk group (Supplementary Figures S1 C–L).

## Discussion

Based on the data from open access public databases, many studies have focused on the link between RNA-seq data of specific genomes and prognosis of individual patients (Sun et al., 2022; Zhao et al., 2022), while few studies were focused on the prognosis of LUAD with specific genomes, particularly on clinical application, immune infiltration and other related areas. There was growing evidence that the response of extracellular matrix to TME drives the potential carcinogenic mechanisms of many cancers, including lung cancer (Li et al., 2021). At present, there were few reports on the prognostic value of BMs-related genes in LUAD. In this study, we have developed a comprehensive model with multi-genes for prediction of prognosis in the patients with LUAD. This study was aimed to investigate the relationship between the expression of BMs-related gene signature and the prognosis of patients with LUAD. We constructed a new prognostic model based on the BMs-based signature which included 10-BMs-related genes, such as ACAN, ADAMTS15, ADAMTS8, BCAN, COL4A3, ITGA8, ITGB4, LAD1, TENM3, and TIMP1. Furthermore, we confirmed the prognostic value of BMs-based signature, and established a survival prediction nomogram involving risk score, age, sex, staging, radiotherapy and chemotherapy, and verified its predictive ability with GSE72094 data sets. In this study, we have demonstrated that the nomogram was verified to have good prediction performance. We further investigated the relationship between BMs-based signatures and clinical features. Finally, we explored the relationship between the differential expression of BMs-related genes and immune checkpoints in patients with LUAD.

In the current study, the risk score was constructed based on 10 BMs-related genes and used to demonstrate its value in clinical research. The ITGB4, LAD1, BCAN and ADAMTS15 were found to have significance in OS of patients with LUAD, of which the integrin subunit β4 (ITGB4) is one of the most characteristic integrins and is involved in regulation of various cellular functions (Giancotti, 2007). Previous studies have shown that integrin regulates angiogenesis, connective tissue proliferation and immune response of tumor host cells by affecting tumor cell migration, invasion, proliferation and survival. Thus it affects epithelialmesenchymal transition (EMT), tumor occurrence, metastasis and even treatment outcome (Xiong et al., 2021). For example, the overexpression of ITGB4 was associated with invasive behavior and poor prognosis of NSCLC (Zheng et al., 2013; Wu et al., 2019). In this study, we found that the high expression of ITGB4 contributes to the poor prognosis of LUAD, which was consistent with previous studies. The LAD1 (Ladinin-1) was a collagen-anchored filament protein on the BM, which was used to maintain the cohesion of the dermis-epidermal junction (Teixeira et al., 2015). It helped to stabilize the connection between the epithelium and the underlying mesenchyme (Motoki et al., 1997). In addition to its structural role, LAD1 also participates in the regulation of mitotic signals by acting as a connexin in EGF-induced ERK5 cascade activation (Yao et al., 2010). Comparative proteomic studies showed that the expression of LAD1 in LUAD was more abundant than that in normal lung tissue (Codreanu et al., 2017). Similarly, we found that the overexpression of LAD1 leads to the poorly prognostic value in the progression of LUAD. The ADAMTS-15 acted as a tumor suppressor in breast and prostate cancer (Porter et al., 2006; Binder et al., 2020). Enhanced expression of ADAMTS-15 might reduce the motor ability of breast cancer cells and angiogenesis, rather than rely on its catalytic activity. Binder et al. found that ADAMTS-15 combined with androgen could inhibit tumor (Binder et al., 2020). However, in our current study, the overexpression of ADAMTS-15 led to a poor prognosis of patients with LUAD. Recent study has found that the expression of BCNA gene can protect bacterial cell capsule from lipid peroxidation free radicals; however, its exact roles in cancer require to be further studied (Naguib et al., 2022). In our study, BCNA was risk factors, and the patients with highexpression had a poor prognosis, which provided evidence for further study in the future.

Our understanding of BMs in normal and disease states remains limited due to the lack of adequate understanding of the role of BM proteins in LUAD. However, in the GO enrichment analysis, we found that BMs was an important part of EMC in the process of BP, CC and MF, which was consistent with previous studies (Baghban et al., 2020; Sahai et al., 2020). The EMC macromolecules exist in all extracellular tissues, coordinating a variety of cellular processes and tumor metastasis (Jayadev et al., 2022). The KEGG analysis showed that BMs-related genes played a significant role in ECM-receptor interaction. The TME, apart from ECM, included fibroblasts, immune cells, and blood vessels. Composition and network organization of EMC were synthesized and modified by cancer-associated fibroblasts (CAFs) and cancer cells (Baghban et al., 2020; Sahai et al., 2020). In this way, the nature of TME is altered, and conversely, the TME can dictate the growth and spread of the tumor. This showed that BMs might play a certain role in the transformation between TME and ECM, which needs futher study to be explored in the future.

Tumor infiltrating lymphocytes (TIL) were indispensable for the occurrence and development of tumors (Kuninty et al., 2022). Although the monotherapy of PD-1 or PD-L1 was generally well tolerated and the efficacy is limited, combination therapy increased the risk of immune-related adverse events. Therefore, new predictive biomarkers were needed to maximize the benefit of patients, reduce toxicity, and guide combination therapy (Ni et al., 2022). We used immune algorithm to find immune checkpoints with differences between high-risk and low-risk groups of BMs. Moreover, LUAD patients in high-risk groups might benefit from immune checkpoint therapy. However, so far, there have been no studies on the association between BMs and drug sensitivity or resistance. Using the CellMine database, we found that the expression of BMs-related genes was related to the sensitivity of Vemurafeni, Dabrafenib, Selumetinib and Cobimetinib, which were targeted drugs for gene mutations. Therefore, we speculated that BMs might play a role in targeted therapy, which might increase the drug sensitivity.

Although this study has found the relationship between BMs-related genes signature and the prognosis of LUAD and clinical significance of prognosis prediction of patients with LUAD, it remains certain limitations. For example, since this study collected the data of LUAD patients from TCGA and GEO public databases and lacked actual laboratory research data, the model was constructed based on such data, the findings are needed to be verified or validated with the real data from the prospectively designed clinical trials.

In conclusion, we determined whether the BMs genes risk characteristics related to the OS of LUAD patients; and constructed and verified the prognostic nomogram of LUAD, including BMs-related risk score, age, sex, stage, radiotherapy and chemotherapy for prediction of individual survival. Moreover, we comprehensively analyzed the differentially expressed BMs-related genes by enrichment analysis, immunity and drug susceptibility. Thus, this study may identify a new BMs-related prognostic marker, demonstrate the clinical significance of BMs in LUAD, and provide some evidence for the future study on the role of BMs in LUAD.

## Data Availability

The original contributions presented in the study are included in the article/Supplementary Material, further inquiries can be directed to the corresponding author.

## Supplementary material

Supplementary Figure S1 Differential expression analysis of immune checkpoints such as the CD276 **(F)**, TNFSF9 **(G)**, CD200R1 **(H)**, CD28 **(I)**, CD80 **(J)**, CD48 **(K)**, TNFS18 **(L)**, TNFS15 **(M)** and CD40LG **(N)** in high- and low-risk groups.

## Abbreviations

BMs, Basement membranes; ECM, Extracellular matrix; LUAD, Lung adenocarcinoma; TCGA, The Cancer Genome Atlas; GEO, Gene expression omnibus; LASSO, Least absolute shrinkage and selection operator; ROC, Receiver operating characteristic; OS, Overall survival; TME, Tumor microenvironment; FDR, False discovery rate; C-index, Concordance index; GO, Gene ontology; MF, Molecular function; BP, Biological processes; CC, Cellular components; KEGG, Kyoto encyclopedia of genes and genomes; PPI, Protein-protein interaction; ITGB4, Integrin subunit β4; EMT, Epithelial mesenchymal transition; NSCLC, Non-small-cell lung cancer; LAD1, Ladinin-1; CAFs, Cancer-associated fibroblasts; TIL, Infiltrating lymphocytes.
