# Supplementary material for: Prognostic roles of a novel basement membranes-related gene signature in lung adenocarcinoma
Source: Front Genet. 2023 Feb 9;14:1100560. doi: 10.3389/fgene.2023.1100560 (PMC9946986; doi:10.3389/fgene.2023.1100560)
Supplement: Supplementary file 1 [file Table1.DOCX]

<https://www.jianguoyun.com/c/sd/1653c6c/1b7c93e657200698>
